# Tobacco control policies in the 21st century: achievements and open challenges

**DOI:** 10.1002/1878-0261.12918

**Published:** 2021-02-15

**Authors:** Armando Peruga, María José López, Cristina Martinez, Esteve Fernández

**Affiliations:** ^1^ Tobacco Control Research Group, Epidemiology and Public Health Research Programme Institut d'Investigació Biomèdica de Bellvitge‐IDIBELL Barcelona Spain; ^2^ Consortium of Centers for Biomedical Research on Respiratory Diseases (CIBERES) Madrid Spain; ^3^ Center for Epidemiology and Health Policies Clínica Alemana School of Medicine Universidad del Desarrollo Santiago Chile; ^4^ Evaluation and Intervention Methods Service Agència de Salut Pública de Barcelona Spain; ^5^ Consortium of Centers for Biomedical Research on Epidemiology and Public Health, CIBERESP Madrid Spain; ^6^ Institut d'Investigació Biomèdica de Sant Pau (IIB Sant Pau) Barcelona Spain; ^7^ Tobacco Control Unit WHO Collaborating Center on Tobacco Control Institut Català d'Oncologia‐ICO Barcelona Spain; ^8^ School of Medicine and Health Sciences Campus of Bellvitge, Universitat de Barcelona Spain

**Keywords:** cancer, global health, health policy, noncommunicable diseases, smoking, tobacco control

## Abstract

Noncommunicable diseases (NCDs), including cancer, are responsible for almost 70% of all deaths worldwide. Tobacco use is a risk factor common to most NCDs. This article discusses tobacco control policies and highlights major achievements and open challenges to reduce smoking prevalence and attributable morbidity and mortality in the 21st century. The introduction of the WHO Framework Convention on Tobacco Control in 2005 has been a key achievement in the field and has already facilitated a drop in both smoking prevalence and exposure to secondhand smoke. Indicatively, the size of the worldwide population benefiting from at least one cost‐effective tobacco control policy has quadrupled since 2007. In addition, plain cigarette packaging has been successfully introduced as a tobacco control policy, surmounting efforts of the tobacco industry to challenge this based on trade and investment law. Nevertheless, tobacco control still faces major challenges. Smoking prevalence needs to be further reduced in a rather expedited manner. Smoke‐free environments should be extended, and the use of plain tobacco packaging with large pictorial health warnings for all tobacco products should be further promoted in some parts of the world. Some of these measures will require prompt determination and diligence. For example, bold political decisions are needed to significantly increase real prices of tobacco products through excise taxes, ban added ingredients that are currently used to increase the attractiveness of tobacco products and ban the tobacco industry's corporate social responsibility initiatives. Finally, the debate on harm reduction strategies for tobacco control still needs to be resolved.

## Graphical abstract

Tremendous, although insufficient, progress has been made on tobacco control during the past twenty years. Nevertheless, there are still open challenges, and several measures remain to be implemented soon: increasing tobacco taxes, banning the use of additives, implementing plain packaging, banning tobacco industry's corporate social responsibility activities, and counteracting the undermining tactics of the tobacco industry.

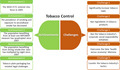

**Abbreviations**
COVID‐19: Corona virus disease 2019
CSR: Corporate Social Responsibility
FCTC: Framework Convention for Tobacco Control
ITP: Illicit Trade Protocol
MPOWER: Monitor tobacco use and prevention policies; Protect people from tobacco smoke; Offer help to quit tobacco use; Warn about the dangers of tobacco; Enforce bans on tobacco advertising, promotion, and sponsorship; and Raise taxes on tobacco
NCD: noncommunicable disease
SDG: Sustainable Development Goal
WHO: World Health Organization
WTO: World Trade Organization

## 1 Introduction

The 1964 US Surgeon General's Report [1] and numerous other reports have established the terrible consequences of smoking on the health of smokers and nonsmokers. At the end of the 20th century, tobacco had caused 100 million deaths worldwide, becoming a leading cause of totally preventable premature deaths. It has been predicted that without any additional tobacco control efforts, one billion people could die from causes related to tobacco by the end of the 21st century, such as cancer, heart disease, stroke, lung diseases, diabetes, and chronic obstructive pulmonary disease [2].

An extremely profitable industry fueled the tobacco epidemic by selling a highly addictive product taking advantage of globalization in the second half of the 20th century. Governments and public health organizations became aware of the globalization and the severe consequences of the tobacco epidemic and its evolution into a large‐scale pandemic [3]. The significant economic toll of tobacco, which today amounts to US$1436 billion, or 1.8% of the world's annual gross domestic product [4], was soon realized. At the same time, governments and public health organizations recognized that the pandemic needed a global and coordinated high‐level response.

**Fig. 1:**
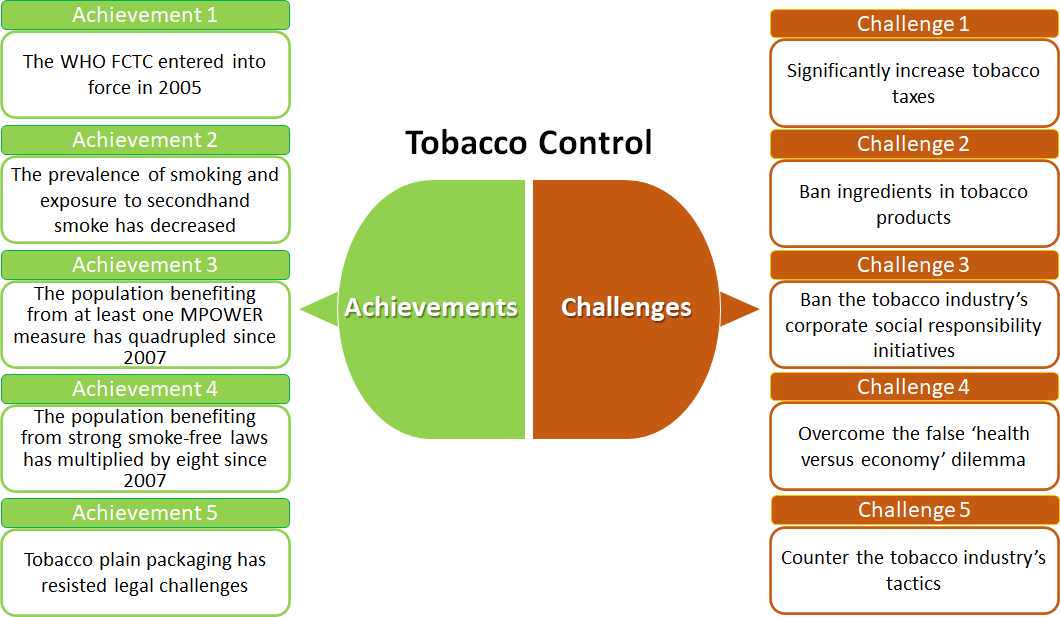
Tremendous, although insufficient, progress has been made on tobacco control during the past 20 years. Nevertheless, there are still open challenges, and several measures remain to be implemented soon: increasing tobacco taxes, banning the use of additives, implementing plain packaging, banning tobacco industry's corporate social responsibility activities, and counteracting the undermining tactics of the tobacco industry.

In 1999, WHO initiated the proceedings to create the Framework Convention for Tobacco Control (FCTC), the first international treaty under WHO auspices. Followingly, the global community recognized tobacco use as a severe threat to global health, as well as a social and economic problem, and began to take joint international action. This work highlights achievements in tobacco control in the 21st century and discusses open challenges (Fig. 1).

## 2 Achievements of tobacco control efforts during the first 20 years of the 21st century

### 2.1 Galvanizing global political will around international law

The WHO FCTC entered into force in 2005 as binding law for all treaty parties. As of January 2021, the treaty was adopted by 181 WHO member states and the European Union, thereby covering more than 90% of the world's population. The Protocol to Eliminate Illicit Trade in Tobacco Products, also known as Illicit Trade Protocol (ITP), was introduced under the WHO FCTC in 2018. As of January 2021, 62 WHO FCTC parties had also become parties to the protocol.

Galvanizing the global political will for implementing the WHO FCTC and the ITP has been a key success in tobacco control. These treaties redefine the role of international law in preventing disease and promoting health. Both treaties seek to establish cooperation among countries to tackle, for example, cross‐border advertising and illicit trade. Importantly, they seek to establish international cooperation on matters that would otherwise be subject to national regulation because the sovereignty of nations to protect public health is often challenged by the interests of the powerful transnational tobacco industry. The tobacco companies often seek to expand the tobacco market through various tactics, including intensive targeting of women, children, and the poorer parts of society [5]. Therefore, the WHO FCTC and the ITP have solidified global governance of health matters and the foundation for countries to enact comprehensive, effective national tobacco control measures that span across all government sectors.

### 2.2 Quadrupling the number of people benefiting from at least one cost‐effective tobacco control policy since 2007

In 2008, WHO introduced the MPOWER package to assist in implementing the six best‐practice cost‐effective interventions defined in the WHO FCTC. The six MPOWER measures are as follows: (a) Monitor tobacco use and prevention policies (M); (b) Protect people from tobacco smoke (P); (c) Offer help to quit tobacco use (O); (d) Warn about the dangers of tobacco (W); (e) Enforce bans on tobacco advertising, promotion and sponsorship (E); and (f) Raise taxes on tobacco (R) (see Table 1 for an overview of MPOWER measures and how they relate to the WHO FCTC provisions) [6]. To track the global improvement in the implementation of MPOWER measures, WHO measures the level of policy achievement for each measure in each country. In each country, an MPOWER measure is considered to be mandated at the highest level when the law requires implementing all policy components that render such measure most efficacious in reducing the demand for tobacco products, that is, reducing the prevalence of tobacco use. For example, the MPOWER measure to protect the population from tobacco smoke is mandated at the highest level when the law requires a complete indoor smoking ban for all workplaces and public places and not only for some of them. Similarly, the measure to warn about the dangers of tobacco is mandated at the highest level when the law requires that health warnings cover an average of at least 50% of the front and back of the package and has four or more desired features. These features include changing the health warning periodically or including pictures or pictograms. Tobacco taxes are mandated at the highest level when excise tobacco taxes amount to at least 75% of the retail price of a cigarette pack. The closer each country is to the highest level of policy achievement, the higher is the MPOWER score the country receives. A detailed description of the MPOWER scores has been explained elsewhere [7].

**Table 1:** Description of the WHO FCTC articles and their inclusion in the MPOWER measures.

Policy topic	FTCT articles	MPOWER measure
General obligations engendered by the treaty	3–5	
Demand‐side reduction measures	6–14	
Increasing price and tax measures as effective means to reduce the demand for tobacco	6	R
Implementing effective measures to protect from exposure to tobacco smoke in indoor workplaces, public transport, indoor public places, and other public places	8	P
Regulating the contents and emissions of tobacco products and disclosing information on their constituents and emissions	9–10	
Banning misleading tobacco packaging and labeling and ensuring that tobacco product packages carry large health warnings and messages describing the harmful effects of tobacco use	11	W
Promoting public awareness of tobacco control issues through all available communication tools	12	W
Banning of all forms of tobacco advertising, promotion, and sponsorship	13	E
Supporting the reduction in tobacco dependence and assisting cessation, including counseling, psychological support, nicotine replacement, and education programs	14	O
Eliminating all forms of illicit trade in tobacco products, prohibiting the sales of tobacco products to or by minors, and supporting economically viable alternative activities to tobacco growing	15–17	
Addressing the severe risks posed by tobacco growing to human health and the environment	18	
Holding the tobacco industry liable for any abuses and promoting cooperation among Parties in legal actions relating to liability	19	
Scientifically and technically cooperating and communicating among Parties, including tobacco control surveillance	20–22	M
Managing institutional arrangements and financial resources of the treaty	23–28	

About 5 billion people living in 136 countries, an equivalent to 65% of the world's population, are currently benefiting from at least one of these MPOWER measures implemented at the highest level. This is a fivefold increase from the 1.1 billion people benefiting from tobacco control measures back in 2007.

The world's population profiting from a basic comprehensive policy to assist smoking cessation, or a comprehensive ban of tobacco advertising, promotion, and sponsorship has increased about sixfold between 2007 and 2018. The proportion of the world's population benefitting from a comprehensive smoke‐free policy or a legal mandate to have large graphic labels with strong health warnings on tobacco packages has increased more than eight times in the same period.

While the increase in cigarette taxes is the most effective tobacco control measure [8], it was also the least applied in 2018. The total population worldwide affected by a cigarette tax representing at least 75% of the retail price has almost doubled since 2007. Another way to look at the impact of tobacco taxes is to assess whether tax increases are able to decrease the affordability of tobacco products. By 2018, 44.3% of the global population lived in countries where cigarettes became less affordable in the last 10 years. However, most decreases in cigarette affordability were small. When considering at least a 10% relative decrease in cigarette affordability, the world's population living in countries achieving this breakthrough is 3.1% [7].

Noticeably, the proportion of the world's population exposed to a best‐practice mass media campaign decreased from 2010 until 2018. Few countries run mass media campaigns regularly, probably due to the high costs of such campaigns. Only four countries (Australia, Turkey, the United Kingdom, and Viet Nam) have run best‐practice mass media campaigns repeatedly since 2010.

### 2.3 Reducing the prevalence of smoking and exposure to secondhand smoke

According to the latest WHO estimates that compared smoking prevalence across countries in 2015, the age‐standardized prevalence of current tobacco smoking had decreased gradually by 5.9 percentage points since the beginning of the 21st century, that is, a relative reduction of 25% or an average decrease of 0.4 percentage points per year. WHO estimates that 19.8% of the world's population aged ≥ 15 years were current smokers in 2015 [9]. Denmark, Norway, and Uruguay were the only countries where current smoking prevalence among persons aged ≥ 15 years had been reduced by ten or more percentage points between 2005 and 2015. During this period, Denmark and Panama approached most closely the endgame prevalence target of 5%, covering more than half of the gap between current smoking prevalence and target [10].

A recent study [11] estimated that in countries with higher initial tobacco control preparedness, as measured by an early MPOWER implementation, the prevalence of daily smoking decreased by between 0.39 and 0.50 percentage points for each increase in the MPOWER score, which indicates the strength of the adopted policies. By contrast, countries with initially low tobacco control preparedness and high daily smoking prevalence seem to be struggling to reduce prevalence despite progress in MPOWER implementation. Another study indicated that the adoption of at least one highest level MPOWER policy in 88 countries between 2007 and 2014 resulted in almost 22 million fewer projected smoking‐attributable deaths [12].

The health impact of smoke‐free policies has been impressive. The proportion of people protected by smoke‐free legislations worldwide has increased from 3.0% in 2007 to 21.1% in 2018 (Table 2). The largest countries in the world report significant decreases in the proportion of people exposed to secondhand smoke [13, 14, 15, 16]. Existing evidence shows that countries that enact national legislative smoking bans reduce the population exposure to passive smoke and benefit from improved health outcomes, specifically of cardiovascular diseases [17].

**Table 2:** Global progress in the implementation of selected tobacco control policies at the highest level^a^. Change between 2007 and 2018 in the population living in countries with selected policy in billions and as a percentage of the world's population.

Policy achievement	2007		2018	
	Billion	%	Billion	%
Total tax on cigarettes ≥ 75% of retail price	0.5^b^	7.6^b^	1.0	13.2
Comprehensive ban of tobacco advertising, promotion, and sponsorship	0.2	3.0	1.3	17.1
Comprehensive smoke‐free policy	0.2	3.0	1.6	21.1
Well‐designed national antitobacco mass media campaigns	2.4^c^	36.4^c^	1.7	22.4
National quitline, and both NRT and some cessation services cost‐covered	0.4	6.1	2.4	31.6
Strong and large graphic health warning on the package	0.4	6.1	3.9	51.3

^a^ The highest level of implementation corresponds to a policy adopted with all the necessary features to make it as effective as possible in achieving its intended goals.

^b^ Year corresponds to 2008.

^c^ Year corresponds to 2010.

### 2.4 Tobacco plain packaging has resisted challenges under trade and investment law

In 2012, Australia became the first country to implement tobacco plain packaging to counter the tobacco industry's use of packaging for both selling cigarettes and undercutting health warnings. The Australian legislation bans logos, brand imagery, symbols, other images, colors, and promotional text on tobacco products and tobacco product packaging. It also requires that graphic health warnings cover 75% of the front and 90% of the back of the tobacco pack [18].

Australia's plain packaging legislation underwent three sets of legal challenges. First, big tobacco companies filed a lawsuit in the Australian High Court. Second, Philip Morris Asia sought to bring down the Australian legislation under an existing investment treaty between Australia and Hong Kong. Third, Cuba, the Dominican Republic, Honduras, Indonesia, and Ukraine filed a dispute through the World Trade Organization (WTO). The constitutional challenge was dismissed in August 2012 [19], and the investment challenge was rejected in December 2015 [20]. The WTO decided in June 2020 that Australia's plain packaging laws are likely to improve public health and that they are not unfairly restrictive to trade [21]. The decisions in the case of Australia are not just a success for public health. They also bring hope for continuing efforts to defend tobacco control policies against the attempts of the wealthy tobacco transnationals.

## 3 Immediate challenges for further reducing the burden to tobacco‐attributable diseases

The successes described above are significant accomplishments. However, key challenges still need to be addressed to reduce the burden of tobacco‐attributable diseases worldwide in a timely manner.

### 3.1 Accelerating the decline of smoking prevalence

The WHO set a relative reduction goal of 30% in tobacco use and smoking for the period between 2010 and 2025 [22]. Accordingly, the global prevalence of current smokers should be 15.1% by 2025. However, based on existing trends, the WHO projects that current smokers would be 17.1% of the global population by 2025 [7]. Therefore, the projected decrease is not fast enough to reach the 2025 reduction goals set by the WHO.

The reduction in smoking prevalence has been, so far, attributed primarily to the increase in the total population and not necessarily to a reduction in the number of smokers. It is projected that the total number of smokers will decrease from 1082 million in 2000 to 1058 million in 2025, a reduction of about 24 million or 2.2% [7]. While the number of smokers in the Americas and Europe will substantially decrease, a net increase in male smokers in the African, Eastern Mediterranean, and South‐East Asian regions is expected to hinder a more significant global decrease. Considering these figures, and that almost one third of the countries of the world—59 countries in total—have not yet adopted any MPOWER measures at the highest level of achievement, the implementation of cost‐effective tobacco control measures needs to be expedited.

Strengthening tobacco denormalization through smoke‐free environments and disseminating plain packaging and large pictorial warnings for all tobacco products could spearhead progress in many countries. It seems, however, that a few measures will require prompt unique determination and diligence. In our opinion, bolder moves are needed to:

- significantly increase real prices of all tobacco products through tobacco taxes. Since increasing taxes is the most effective tobacco control measure, the tobacco industry devotes many efforts to derail this measure [23, 24]. The main tactics employed by these companies depend on the tax structure and administration of each country and the type of competition they face from other manufacturers [25].
- disrupt strategies currently applied to engineer the attractiveness of tobacco products by banning ingredients that may increase their palatability, including additives and particularly characterizing flavors.
- ban the most insidious form of tobacco promotion: the tobacco industry's corporate social investment or responsibility (CSR) initiatives. The tobacco industry has always conceived CSR as a public relations tool to further its business objectives [26]. It is a form of advertising, promotion, and sponsorship that should be banned. Whether supporting empowering women [27], disaster relief and preparedness [28], infectious disease prevention [29], or efforts against COVID‐19 [30], the tobacco industry's CSR activities do little to address the death and suffering caused by tobacco use [31].

To accelerate the implementation of these and other measures and the decline of smoking, some consider that a harm reduction strategy should be added to the existing mix of policies. A harm reduction approach to tobacco control encourages those smokers that cannot or are unwilling to stop smoking to switch to using nicotine in a less harmful form than combustible tobacco [32]. The public health community is divided over the value of such a strategy within the parameters of the existing alternative products, market forces driving the use of all tobacco and nicotine products, the strength of tobacco control policies, and the room of these to significantly and quickly drive a reduction in smoking [33]. Resolving this debate is a challenge too. Meanwhile, there are at least three things that should be considered to expedite the implementation of the WHO FCTC, as discussed below.

### 3.2 Positioning tobacco control in the global health and development agendas

The global success of the WHO FCTC will be partially determined by the extent to which governments and the international community realize that the tobacco pandemic is a threat to development and the achievement of the United Nations Sustainable Development Goals (SDG) [34]. Tobacco use increases healthcare costs and decreases productivity. Moreover, it feeds into the vicious circle of poverty. The most disadvantaged people spend comparatively less on necessities such as food, education, and health care to pay for their addiction to tobacco products [35]. Furthermore, tobacco farming destroys the environment upon which the poorest rely to survive. The large amounts of pesticides and fertilizers required to grow tobacco are toxic and pollute water supplies, in addition to the deforestation of their habitat to make room for a nonstaple crop and to cure tobacco [36]. Despite the inclusion of a specific target for implementing the WHO FCTC in the SDGs, for most governments, tobacco control remains merely a health issue instead of a development goal [37].

Noncommunicable diseases presently make up 7 of the world's top ten causes of death, and tobacco use is a risk factor for many NCDs [38]. However, tobacco control is often not prioritized in the health policy agenda [32]. The global health agenda is presently dominated by the ‘unfinished agenda’ of communicable disease and maternal and child health in low‐ and middle‐income countries. Considering the threats of tobacco use to the public health systems, tobacco control's contribution to building stronger economies and more equitable societies will help to address the ‘unfinished agenda’ and will be crucial for the recovery from the COVID‐19 pandemic in low‐ and middle‐income countries [39].

The exposure of high‐income countries to the COVID‐19 pandemic has highlighted the importance of controlling communicable diseases also in these nations. However, this should not distract us from the fact that COVID‐19 has hit the hardest people with NCDs, for which tobacco use is the main common risk factor. Smoking increases the risk of hospitalization, disease severity, and mortality from COVID‐19 [40]. Therefore, the COVID‐19 pandemic highlights the importance of investing equal efforts in tackling communicable diseases and NCDs, as the latter impact on the health outcomes of the former, as well as on the capacity of healthcare systems.

Tobacco control, and NCD prevention, in general, involves the regulation of industries that produce goods whose consumption may affect human health. Some of these industries and their allies are self‐servingly reminding us that the priority for global health is to prevent communicable disease [41] and responding quickly and decisively to outbreaks [42] instead of tobacco control or NCDs [43, 44].

### 3.3 Overcoming the false ‘health *versus* economy’ dilemma: the need for a whole‐government approach

The response to the COVID‐19 pandemic has made us painfully aware of the fallacy of presenting the response to health problems as a trade‐off between lives saved and the economic cost of trying to save those lives—the health *versus* the economy dilemma. Positioning tobacco control within the overall—mainly economic—priorities of each government is a challenge, mainly given the intricacies of the broader context of the economic globalization that governments must navigate.

Parties to the WHO FCTC recognize that a critical challenge to implementing the treaty in their countries is the weakness of their multisectoral coordination and the insufficient support to the implementation of the WHO FCTC from sectors outside health [45]. A whole‐government approach is needed to succeed in declining smoking prevalence.

### 3.4 Countering the Tobacco Industry's Tactics to undermine tobacco control measures

The interests of the tobacco industry are irreconcilable with tobacco control and public health [46]. Consequently, governments should protect the implementation of their tobacco control policies from the commercial and other vested interests of the tobacco industry as mandated by the WHO FCTC. Countering the tobacco industry's tactics to undermine tobacco control measures is not a new challenge [47], but it has evolved with time. From the same that claimed at some point that tobacco is not damaging to health [48], nor addictive [49] or denied targeting youth [50], we get now that they are committed to a ‘smoke‐free future’ [51]. Their claims are not credible as long as the industry continues to fight proven policies and programs that reduce smoking. Equally, their proclamations are not convincing while they misrepresent regulatory agency decisions about the novel tobacco products such as heated tobacco products as less harmful than cigarettes [52]. Ultimately, if anyone in the tobacco industry is really dedicated to a smoke‐free future, it should immediately stop all marketing of any kind of cigarettes.

## 4 Conclusions

Tremendous, although insufficient, progress has been made on tobacco control during the past twenty years (Fig. 1). Nevertheless, there are still open challenges, and several measures remain to be implemented soon: increasing the real price of all tobacco products through tobacco taxes, banning the use of additives in tobacco products, implementing plain packaging for all tobacco products, and banning tobacco industry's corporate social responsibility activities. While implementing these measures, governments and public health policymakers should be prepared to counteract undermining tactics of the tobacco industry.

## Conflict of interest

The authors declare no conflict of interest.

## Author contributions

AP, MJL, CM, and EF contributed to the conception and outline of the manuscript. AP prepared the first version of the manuscript. AP, MJL, CM, and EF edited and revised the manuscript, and approved its final version.
